# Expression profiles and potential functions of long non-coding RNA in stable angina pectoris patients from Uyghur population of China

**DOI:** 10.1042/BSR20190364

**Published:** 2019-09-03

**Authors:** Xin-Rong Zhou, Ning Song, Jun-Yi Luo, Hui Zhai, Xiang-Mei Li, Qian Zhao, Fen Liu, Xiao-Mei Li, Yi-Ning Yang

**Affiliations:** 1 Department of Cardiology, First Affiliated Hospital of Xinjiang Medical University, Urumqi, Xinjiang, China; 2 Xinjiang Key Laboratory of Cardiovascular Research, Urumqi, Xinjiang, China

**Keywords:** large intervening non-coding RNA, microarray, stable angina pectoris

## Abstract

Long non-coding RNAs (lncRNAs) are transcripts longer than 200 nt that are involved in cardiovascular diseases (CVDs). To determine whether lncRNAs are involved in stable angina pectoris (SAP), we analysed the expression profile of lncRNAs and mRNAs on a genome-wide scale in SAP of Uyghur population. Five pairs of SAP patients and healthy controls were screened by an Agilent microarray (human lncRNA + mRNA Array V4.0). Quantitative real-time polymerase chain reaction (qRT-PCR) was used to validate the lncRNA expression levels in 50 SAP and 50 controls. Data analyses were performed using R and Bioconductor. A total of 1871 up- and 231 down-regulated lncRNAs were identified to be differentially expressed in the peripheral blood mononuclear cells (PBMCs). Microarray analysis results identified the lncRNAs NR_037652.1, ENST00000607654.1, ENST00000589524.1 and uc004bhb.3, which were confirmed by qRT-PCR. Among screened lncRNAs, the annotation result of their co-expressed mRNAs showed that the most significantly related pathways were the NF-κB signalling pathway, apoptosis and the p53 signalling pathway, while the main significantly related diseases were the cholesterol, calcium and coronary disease. Our study indicated that clusters of lncRNAs were significantly differentially expressed between SAP patients and matched controls. These lncRNAs may play a significant role in SAP development and could serve as biomarkers and potential targets for the future treatment of SAP.

## Introduction

Stable angina pectoris (SAP) is one of the most common manifestations of coronary heart disease (CHD) and is associated with myocardial ischaemia due to the increased oxygen requirement and decreased diastolic perfusion time [1,2]. Although the annual mortality rate due to SAP is relatively low [3,4], but it seriously affects the prognosis and quality of life for CHD patients. Conventional treatment strategies, such as medication, revascularisation and lifestyle modification, have often been used for SAP patients [5,6], however, these treatments do not completely relieve angina-related symptoms for most patients. Moreover, there is a lack of knowledge regarding multiple conventional risk factors and serum biomarkers for SAP diagnosis, gene expression profiling using microarrays has promoted the development of many novel molecular biomarkers [7,8].

Long non-coding RNAs (lncRNAs) are a class of well-studied non-coding RNAs that are >200 nucleotides long and lack protein-coding capability [9]. Recently, lncRNAs have emerged as powerful regulators of biological processes, including RNA–RNA interactions and epigenetic and post-transcriptional regulation [10]. Functionally, many studies have demonstrated that lncRNAs play indispensable roles in the pathophysiologic process of CHD [11,12]. For example, the expression of the lncRNA ANRIL is associated with the risk of coronary atherosclerosis [13], the expression of the lncRNA MIAT in peripheral blood mononuclear cells (PBMCs) is significantly reduced in patients with ST segment elevation myocardial infarction (STEMI) [14], and the lncRNA LIPCAR is down-regulated after acute myocardial infarction (AMI) but is increased during later stages of heart failure [15]. The Chinese Uyghur population, a Muslim minority, accounts for 48.53% of the population in Xinjiang. The Uyghur population has a unique lifestyle with a high incidence of CHD [16]. To date, the genome-wide expression of lncRNAs and their potential biological functions in Uyghur SAP patients remain unknown.

To further identify the potential lncRNAs markers for SAP, we collected PBMCs from five SAP patients and five matched controls to identify the different expression profiles of lncRNAs and mRNAs among SAP and healthy control. The co-expression relationships among the target genes which confirmed the differentially expressed lncRNAs were analyzes via a regulatory co-expression network. Overall, the present study identified differentially expressed lncRNAs and their potential corresponding mRNAs to predict SAP and to provide a basis for the study of the pathogenesis of SAP.

## Materials and methods

### Patients and samples collection

All patients were evaluated at the time of diagnostic cardiac catheterisation and coronary angiography. In all cases, at least one haemodynamically significant (≥50%) stenosis was present in a major coronary artery [2]. Coronary angiograms were obtained at the Catheter Laboratory in the Department of Cardiology at the First Affiliated Hospital of Xinjiang Medical University. To weaken the variation between samples as much as possible, blood samples from no coronary stenosis matched with age and sex were used as controls. A history of congestive heart failure, unstable angina pectoris (UAP) and MI, severe hepatic or renal dysfunction, malignant tumour, recent infection or active chronic inflammatory disease during the last 6 weeks was excluded in all participants. Five SAP patients (SAP-U2, SAP-U3, SAP-U6, SAP-U7, SAP-U8) and five matched controls (N-U2, N-U3, N-U4, N-U5, N-U6) were randomly selected for lncRNA chip analysis. Blood samples from the rest of 50 SAP patients and 50 controls were obtained to validate the lncRNA expression level using quantitative real-time polymerase chain reaction (qRT-PCR). Additionally, blood samples (5 ml) were collected from peripheral veins before the administration of any anticoagulants, and put into test tubes containing EDTA.

### Collection and purification of PBMCs

Blood samples within 2 h after harvesting were centrifuged at 3000 rpm for 10 min, then divided into two layers. The upper plasma layer was discarded, and the lower layer was used for lysis reaction. Lymphocyte Separation Medium (twice the total blood volume) (TBD, Tianjin Biotechnology) was then added to start the lysis reaction, after mixing gently and then centrifuged at 1500 rpm for 20 min. Thereafter, a white membrane, that is the white cell layer, was transferred to a new tube and centrifuged at 10000 rpm for 5 min. After removal of the supernatant, the PBMCs were left in tubes.

### RNA extraction, labelling and hybridisation

Total RNA, including lncRNAs, was extracted using TRIzol reagent (Invitrogen) and then purified according to the manufacturer’s instructions using a mirVana miRNA Isolation Kit (Ambion, Austin, TX, U.S.A.). Total RNA and OD_260/280_ readings were quantified using a Nanodrop-2000 (Thermo Fisher Scientific, Waltham, MA). The RNA quality and the amount of lncRNAs were measured using an Agilent Bioanalyzer (Agilent Technologies, CA). Sample labelling and array hybridisation were performed according to the Agilent One-Color Microarray-Based Gene Expression Analysis Protocol (Agilent Technology). In brief, Cy5 and Cy3-dCTP were used to label cDNA. Double-stranded cDNAs containing the T7 RNA polymerase promoter were synthesised with T7 Oligo (dT) and T7 Oligo (dN) primers using CbcScript reverse transcriptase (Capitalbio). The dsDNA products were purified using a PCR NucleoSpin Extract II Kit (MN) and eluted with 30 μl of elution buffer for transcription reactions using T7 Enzyme Mix. The amplified cRNA was purified using an RNA Clean-up Kit (MN). Then, a Klenow enzyme labelling strategy [17] was used; 5 μl of Klenow buffer, dNTPs, and Cy5-dCTP or Cy3-dCTP (GE Healthcare) were added, and the mixture was incubated at 37°C for 90 min. Before loading on to a microarray, hybridisation solution including DNA was denatured at 95°C for 3 min. The arrays were hybridised overnight at a rotation speed of 20 rpm in an Agilent Hybridization Oven at 42°C and then washed consecutively with two different solutions (2× saline sodium citrate (SSC) for 5 min at 42°C and 0.2× SSC with 0.2% sodium dodecyl sulphate (SDS) for 5 min at room temperature).

### LncRNA expression profiles

Approximately 200 ng of RNA from each sample was applied for the lncRNA microarray analysis. The lncRNA expression profiles were analysed using an Agilent Human lncRNA + mRNA Array V4.0 (4 × 180K format) that contained 41000 human lncRNA probes and approximately 34000 human mRNA probes. The lncRNAs and their mRNA target sequences were obtained from multiple databases, including GENCODE/ENSEMBL, LNCipedia, the Human LincRNA Catalog, the ncRNA Expression Database (NRED), RefSeq, the University of California, Santa Cruz (UCSC), and the Chen Ruisheng laboratory (Institute of Biophysics, Chinese Academy of Science). Each RNA was detected two times by probes.

### Microarray imaging and data analysis

Data summarisation, normalisation and quality control concerning the lncRNA + mRNA array data were performed in GeneSpring software V13.0 (Agilent). Volcano plot filtering and hierarchical clustering were used to identify the differentially expressed lncRNAs and mRNAs. Differentially expressed genes with statistical significance were identified with a random variance model, and the *P*-values were determined using paired *t* tests. Classification of genes as up- and down-regulated required a fold-change (Fc) >2.0 and a *P*<0.05. A tree was constructed with Java TreeView (Stanford University School of Medicine, Stanford, CA, U.S.A.).

### Gene Ontology and Kyoto Encyclopedia of Genes and Genomes analyses

Gene Ontology (GO) analysis was undertaken to illustrate the unique biological significance of the differentially expressed genes [18]. GO categories describe potential functions related to three defined terms: biological processes, molecular functions and cellular components. Kyoto Encyclopedia of Genes and Genomes (KEGG) analysis was carried out to identify crucial pathways related to gene maps based on the latest KEGG database. The significant GO terms and pathways were identified by Fisher’s exact tests and Chi-square tests, and the significance threshold was defined by false discovery rate (FDR) and *P*-value [19].

### Construction of the regulatory co-expression network

It is important to accurately understand the biological functions of co-expressed proteins, which are important for identifying novel and significant genes. For each pair of genes, a Pearson correlation coefficient was calculated and the significant correlation pairs were selected for construction of the network [8]. The search tool for the open-source bioinformatics software Cytoscape [20] is a reliable online tool that can be used to evaluate many co-expression relationships. In the present study, lncRNAs and mRNAs with Pearson correlation coefficients of at least 0.99 were selected to draw a network with Cytoscape. In a network analysis, a degree is the simplest and most important measure of a gene centrality within a network that determines its relative importance [21].

### Validation of lncRNAs by qRT-PCR

The expression of four lncRNAs was validated by qRT-PCR. cDNA was synthesised by reverse transcription from RNA with an RNeasy Mini Kit (QIAGEN, China) in accordance with the manufacturer’s protocol. Then, qRT-PCR was carried out using Power SYBR Green PCR Master Mix (Applied Biosystems, U.S.A.) in a 7900 HT Fast Real-Time PCR system (Applied Biosystems, U.S.A.). Primers for NR_037652.1, ENST00000607654.1, ENST00000589524.1 and uc004bhb.3, and GAPDH were synthesised by Invitrogen (Shanghai, China). All of the primer sequences used are shown in Supplementary Table S1. To quantify the results, the expression of each lncRNA was calculated using the 2^−ΔΔ*c*^_t_ method.

**Table 1:** Clinical characteristics of five Uyghur SAP patients and five Uyghur matched controls

Characteristics	Controls (*n*=5)	SAP patients (*n*=5)
Age, years	51.1 ± 2.14	51.6 ± 2.83
Gender (male/female)	3/2	3/2
BMI	23.7 ± 2.02	24.9 ± 2.24
Hypertension (Yes/No)	0/5	0/5
Arrhythmia (Yes/No)	0/5	0/5
Diabetes (Yes/No)	0/5	0/5
Smoking (Yes/No)	1/4	1/4
Drinking (Yes/No)	1/4	1/4
SBP, mmHg	120.1 ± 7.24	120.9 ± 8.35
DBP, mmHg	82.4 ± 7.25	81.6 ± 9.49
Heart rate, beats per minute	71.6 ± 4.84	75.9 ± 6.86
Serum creatinine, μmol/l	80.7 ± 16.79	77.9 ± 17.49
FPG, mmol/l	4.89 ± 0.41	5.56 ± 1.16
TG, mmol/l	1.79 ± 0.5*	2.94 ± 1.97*
TC mmol/l	4.57 ± 0.77	4.97 ± 0.86
LDL, mmol/l	3.11 ± 0.91	2.97 ± 0.83
Serum calcium, mmol/l	2.47 ± 0.11	1.94 ± 0.65

The data are presented as the mean ± standard deviation. Abbreviations: BMI, body mass index; DBP, diastolic blood pressure; FPG, fasting plasma glucose; LDL, low-density lipoprotein; SBP, systolic blood pressure; TC, total cholesterol; TG, triglyceride. *P<0.05 compared with healthy controls.

### Statistical analysis

The data are presented as the mean ± standard deviation (SD) or standard error (SEM). Continuous variables were compared using either the Mann–Whitney U test or the Kruskal–Wallis test. For categorical variables, either the Chi-square test or Fisher’s test was used as appropriate. GO and KEGG analyses were evaluated using Fisher’s exact test. For all statistical analyses, IBM SPSS Statistics 18.0 software (SPSS Inc, Chicago, IL, U.S.A.) was used, and a two-tailed *P*<0.05 was considered to be significant.

## Results

### Patient enrolment

The lncRNA and mRNA expression profiles in five SAP patients and five matched healthy controls were detected using a microarray, and 50 SAP patients and 50 matched controls were used for clinical validation. The clinical characteristics of ten participants for lncRNA chip analysis are shown in Table 1.

### Differentially expressed lncRNAs and categorisation between SAP patients and healthy controls

**Figure 1:**
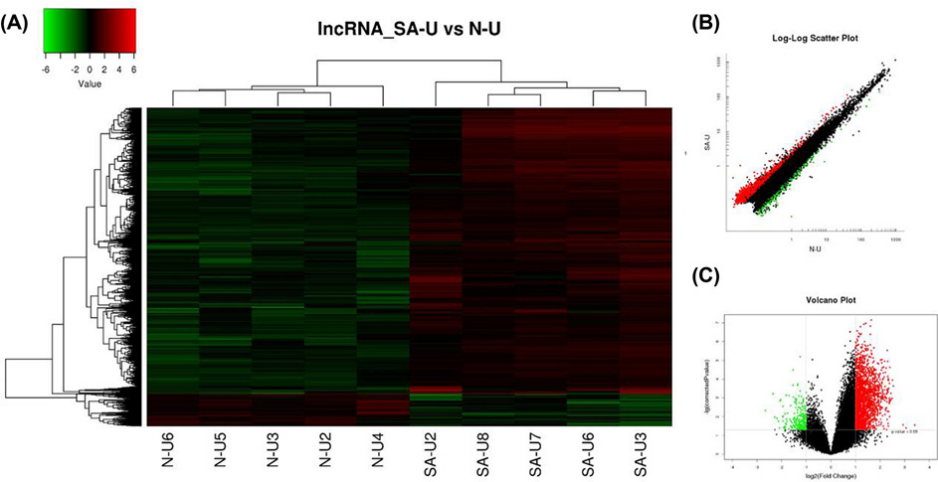
Differential expression of lncRNAs in SAP patients and control individuals of Uyghur ethnicity (**A**) Expression values are represented through hierarchical clustering analysis in red and green, indicating up- and down-regulated lncRNA expressions in SAP patients (SAP-U2, SAP-U3, SAP-U6, SAP-U7, SAP-U8) or control individuals (N-U2, N-U3, N-U4, N-U5, N-U6), respectively. (**B**) Scatter plot of differential lncRNA expression. x-axis: N-U, y-axis: SAP-U. (**C**) Volcano plot of differential lncRNA expression. The red and green spots indicate up- and down-regulation, respectively. x-axis: log2 Fc; y-axis: −1 × log10 (corrected *P*-value) for each probe.

In total, 2102 lncRNAs displayed differential expression between two groups using the criteria of a corrected *P*<0.05 and an Fc > 2.0, including 1871 up-regulated and 231 down-regulated lncRNAs. A selection of lncRNAs showing distinctive expression in Uyghur SAP patients are presented in Table 2. As hierarchical clustering represents one of the simplest and most widely used techniques, we used this analysis which can then enable the generation of hypotheses about the relationships among samples in our experiment. The results of hierarchical clustering showed distinguishable lncRNA expression profiles between the two groups (Figure 1A). Scatter and volcano plots were also used to assess the variations in gene expression between two groups (Figure 1B,C).

**Table 2:** Top 20 differentially expressed lncRNAs in SAP patients in Uyghur based on Fc values

lncRNA ID	Probe name	Fc (abs)	Regulation	Chrom	Class	Database
ENST00000471935.1	p15135	6.2932	Down	7	Intergenic	ENSEMBL
TCONS_00015557	p24545	5.070091	Down	9	Divergent	HumanLincRNACatalog
ENST00000443162.1	p15157	4.84097	Down	7	Antisense	ENSEMBL
uc001vjh.1	p25856	4.54429	Down	13	Intronic	UCSC
ENST00000584157.1	p6780	4.36858	Down	17	Intronic	ENSEMBL
XR_158932.2	p30363	4.172306	Down	19	Divergent	RefSeq
ENST00000529252.2	p29496	4.092166	Down	8	Antisense	ENSEMBL
ENST00000533528.1	p2878	3.868413	Down	11	Divergent	ENSEMBL
ENST00000526377.1	p2591	3.836865	Down	11	Intergenic	ENSEMBL
ENST00000425554.1	p913	3.818452	Down	1	Divergent	ENSEMBL
int-HOXA3-11	p28119	10.6204	Up	7	Intergenic	HOX Loci
ENST00000558536.1	p5543	8.313788	Up	15	Antisense	ENSEMBL
TCONS_00011823	p23452	7.638344	Up	6	Divergent	HumanLincRNACatalog
ENST00000597915.1	p34744_v4	5.823942	Up	2	Antisense	ENSEMBL
ENST00000534297.1	p2546	5.668468	Up	11	Antisense	ENSEMBL
ENST00000536100.1	p3485	5.616653	Up	12	Antisense	ENSEMBL
LIT2106	p33935	5.426726	Up	10	Antisense	RNAdb
TCONS_00029064	p21721	5.422044	Up	21	Intergenic	HumanLincRNACatalog
TCONS_00022290	p29659	5.31662	Up	13	Intergenic	HumanLincRNACatalog
TCONS_00020945	p29634	5.266935	Up	12	Intergenic	HumanLincRNACatalog

**Figure 2:**
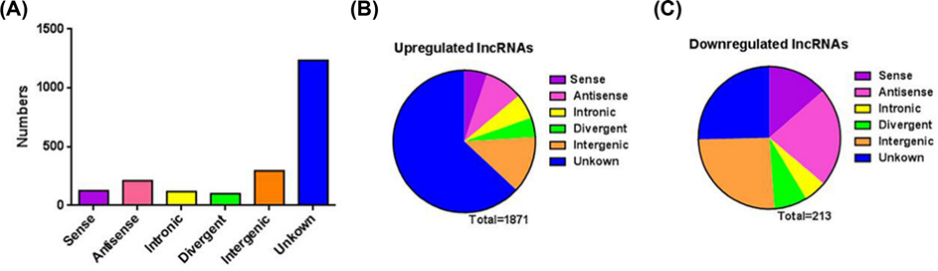
The classification of lncRNAs according to their correlations with protein-coding genes (**A**) The numbers of identified lncRNAs in six categories. (**B**) Pie chart showing the number of up-regulated lncRNAs according to criteria, Fc > 2 and *P*<0.05 in each category. (**C**) Pie chart showing the number of down-regulated lncRNAs according to criteria, Fc > 2 and *P*<0.05 in each category.

**Figure 3:**
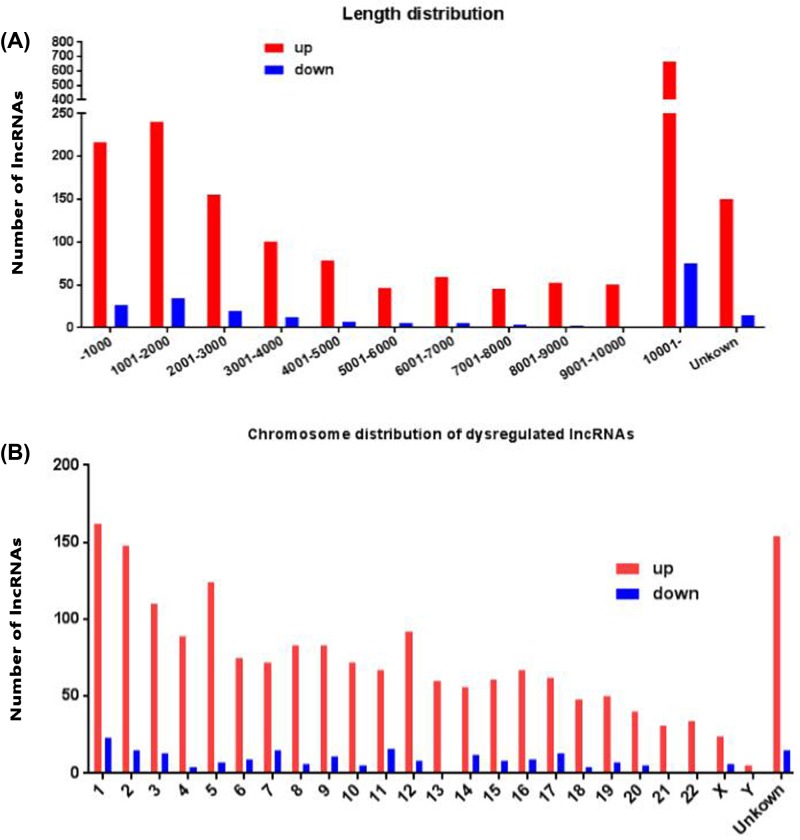
The length and chromosomes distribution of dysregulated lncRNAs (**A**) The length distribution of dysregulated lncRNAs. x-axis: length distribution, y-axis: number of lncRNAs. (**B**) Chromosomes distribution of up- and down-regulated lncRNAs location. x-axis: chromosomes distribution, y-axis: number of lncRNAs.

Next, we analysed distinctive lncRNAs based on their categorisations (Figure 2A). Although 59.21% of the lncRNAs were not successfully categorised, the well-annotated lncRNAs were classified into five categories: 14.20% were intergenic, 10.08% were antisense, 5.66% were intronic, 6.09% were sense and 4.75% were bidirectional. This pattern was also present regarding both the up- and down-regulated lncRNAs (Figure 2B,C). The lengths of the dysregulated lncRNAs were mostly between 200 and 3000 bp (Figure 3A). The chromosome distribution shows the number of up- and down-regulated lncRNAs located within each chromosome (Figure 3B). Up-regulated lncRNAs were mainly located in chr1, chr2, chr3, chr5 and unknown chromosome groups, and down-regulated lncRNAs were mainly located in chr1, chr2, chr3, chr7, chr11 and unknown chromosome groups.

### mRNA expression profiles between SAP patients and healthy controls

**Figure 4:**
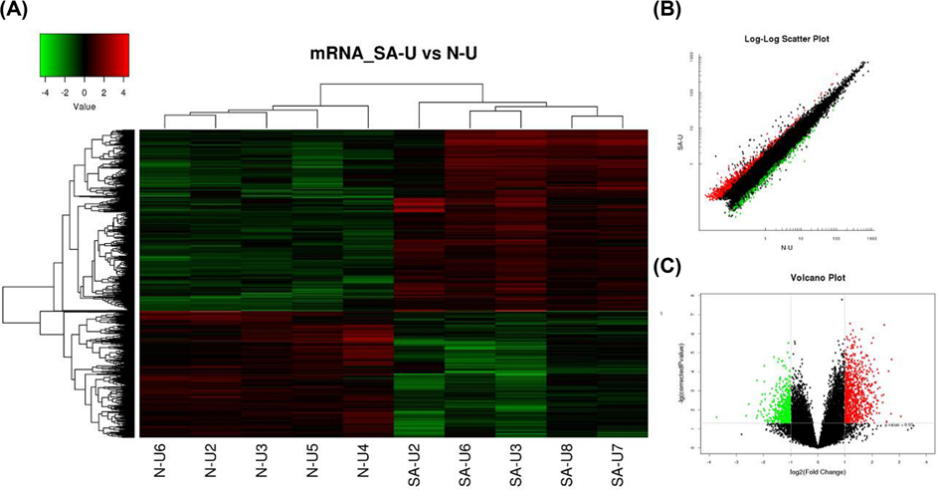
Differential expression of mRNAs in SAP patients and control individuals of Uyghur ethnicity (**A**) Expression values are represented through hierarchical clustering analysis in red and green, indicating up- and down-regulated mRNA expressions in SAP patients (SAP-U2, SAP-U3, SAP-U6, SAP-U7, SAP-U8) or control individuals (N-U2, N-U3, N-U4, N-U5, N-U6), respectively. (**B**) Scatter plot of differential mRNA expression. x-axis: N-U, y-axis: SAP-U. (**C**) Volcano plot of differential mRNA expression. The red and green spots indicate up- and down-regulation, respectively. x-axis: log2 Fc; y-axis: −1 × log10 (corrected *P*-value) for each probe.

As the function of most of these 2102 differentially expressed lncRNA probes remains unknown, we predicted their potential functions through annotation of their co-expressed mRNAs. Among the 34000 detected mRNA probes, a total of 1349 were found to be significantly differentially expressed between SAP patients and healthy controls (Figure 4A). Of these 1349 probes, 795 were up-regulated and 554 were down-regulated. A partial summary of the distinctively expressed mRNAs in Uyghur SAP patients is presented in Table 3. The scatter and volcano plots generated from these differentially expressed probes are clearly segregated between two groups clusters (Figure 4B,C). This result suggests that these mRNAs were substantially different between the SAP and healthy controls.

**Table 3:** Top 20 differentially expressed mRNAs in SAP patients in Uyghur based on Fc values

Probe name	*P*	FC (abs)	Regulation	Gene symbol	Ensembl ID
A_21_P0004887	0.022326836	8.341828283	Up	lnc-CD2AP-2	Unknown
A_23_P216556	2.24801E-05	6.589254801	Up	EPB41L4B	ENST00000374557
A_21_P0003860	0.01624716	6.498708617	Up	LOC101928223	ENST00000504509
A_33_P3251896	9.39614E-05	6.088366922	Up	APBB2	ENST00000543538
A_33_P3280561	0.044000295	5.807894799	Up	KRTAP16-1	ENST00000391352
A_23_P74609	0.041502611	5.779144021	Up	G0S2	ENST00000367029
A_21_P0014105	3.50089E-07	5.46040203	Up	LOC101927206	Unknown
A_23_P415541	0.001620401	5.376366065	Up	GPR26	ENST00000284674
A_23_P6596	0.004156197	5.246830347	Up	HES1	ENST00000476918
A_21_P0008258	0.001797978	5.140095227	Up	LINC00376	ENST00000439454
A_33_P3294720	0.024651055	13.271854	Down	LOC100130865	Unknown
A_23_P217917	0.020856044	6.223089456	Down	GSTM4	ENST00000336075
A_33_P3238579	0.005048665	5.830537859	Down	Unknown	ENST00000611787
A_33_P3424067	0.026767724	4.778261734	Down	SUV420H1	ENST00000615954
A_23_P319874	0.000290347	4.725299189	Down	TCAIM	ENST00000396078
A_23_P407565	0.020509796	4.675297736	Down	CX3CR1	ENST00000541347
A_24_P79617	0.011647027	4.560089019	Down	KIAA0040	ENST00000423313
A_24_P930111	0.005143622	4.327681889	Down	SLC4A10	ENST00000446997
A_24_P276490	0.013011988	4.290633557	Down	LYPLA2	ENST00000420982
A_24_P6903	0.00363308	4.218718187	Down	ACTBL2	ENST00000423391

### Identification of potentially functional mRNAs in SAP patients

**Figure 5:**
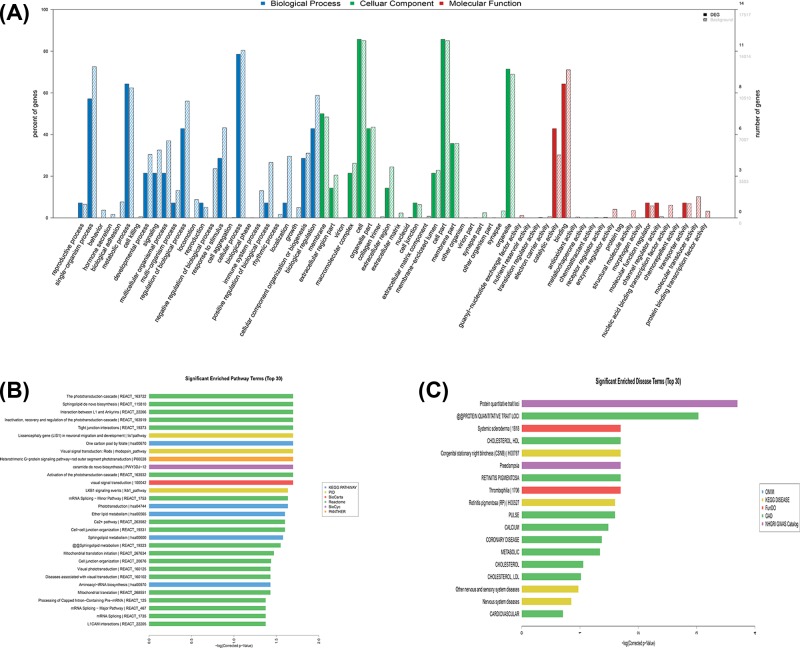
GO, KEGG pathways and Disease analyses of differentially expressed mRNAs (**A**) GO analysis of differentially expressed mRNAs. The top 30 significantly enriched GO categories were determined and were plotted as −1 × log10 (*P*-value). (**B**) KEGG pathways of differentially expressed mRNAs. The top 30 significantly enriched KEGG categories were determined and were plotted as −1 × log10 (*P*-value). (**C**) Disease analysis of differentially expressed mRNAs. The top 30 significantly enriched disease categories and pathways were determined and were plotted as −1 × log10 (*P*-value).

Among the GO terms enriched among the differentially expressed mRNAs in SAP patients, the top ten terms associated with biological processes included: (1) single-organism processes, (2) metabolic processes, (3) regulation of biological processes, (4) responses to stimuli, (5) cellular processes, (6) cellular component organisation or biogenesis, (7) biological regulation, (8) developmental processes, (9) signalling and (10) multicellular organismal processes. The top ten GO terms related to cellular components in the SAP patients included the following: (1) membrane, (2) cell, (3) organelle, (4) membrane-enclosed lumen, (5) organelle part, (6) extracellular region, (7) cell part, (8) extracellular region part, (9) macromolecular complex and (10) membrane part. The top ten GO terms related to molecular functions in SAP patients included: (1) antioxidant activity, (2) enzyme regulator activity, (3) structural molecule activity, (4) morphogen activity, (5) molecular function regulator, (6) channel regulator activity, (7) nucleic acid binding transcription factor activity, (8) transporter activity, (9) molecular transducer activity and (10) protein-binding transcription factor activity (Figure 5A).

Based on KEGG analysis, the most enriched pathways corresponding to the dysregulation of mRNAs related to SAP were the phototransduction cascade; inactivation, recovery and regulation of the phototransduction cascade; heterotrimeric G-protein signalling pathway-rod outer segment phototransduction; activation of the phototransduction cascade; and visual phototransduction (Figure 5B). The top ten significantly enriched disease terms included: (1) protein quantitative trait loci, (2) systemic scleroderma, (3) cholesterol and HDL, (4) congenital stationary night blindness, (5) preeclampsia, (6) retinitis pigmentosa, (7) thrombophilia, (8) pulse, (9) calcium and (10) coronary disease (Figure 5C).

**Figure 6:**
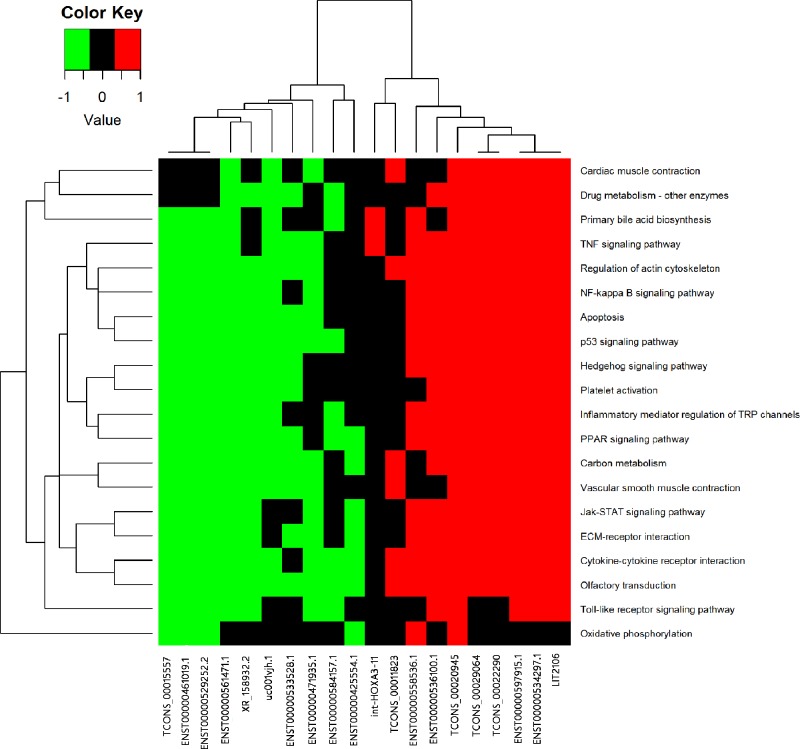
KEGG annotation for the top 20 lncRNAs via co-expression of their mRNA functions Red shows higher expression, green shows lower expression, and the black indicates no relationship.

In the fact, the GO terms and KEGG analysis do not refer to lncRNAs directly, so it is necessary to draw the relationship intuitively. In the Figure 6, the hierarchical clustering figure presents a more intuitive tool to see the relationship between the top 20 lncRNAs and pathways through enrichment analysis of the co-expression of mRNA in microarray analysis, which were related to cardiac muscle contraction, drug metabolism-other enzymes, primary bile acid biosynthesis, the TNF signalling pathway, regulation of the actin cytoskeleton, the NF-κB signalling pathway, apoptosis, the p53 signalling pathway, the hedgehog signalling pathway, platelet activation, inflammatory mediator regulation of TRP channels, the PPAR signalling pathway, carbon metabolism, vascular smooth muscle contraction, the Jak-STAT signalling pathway, ECM–receptor interaction, cytokine–cytokine receptor interaction, olfactory transduction, the Toll-like receptor signalling pathway and oxidative phosphorylation (Figure 6).

### LncRNA–mRNA network analysis

**Figure 7:**
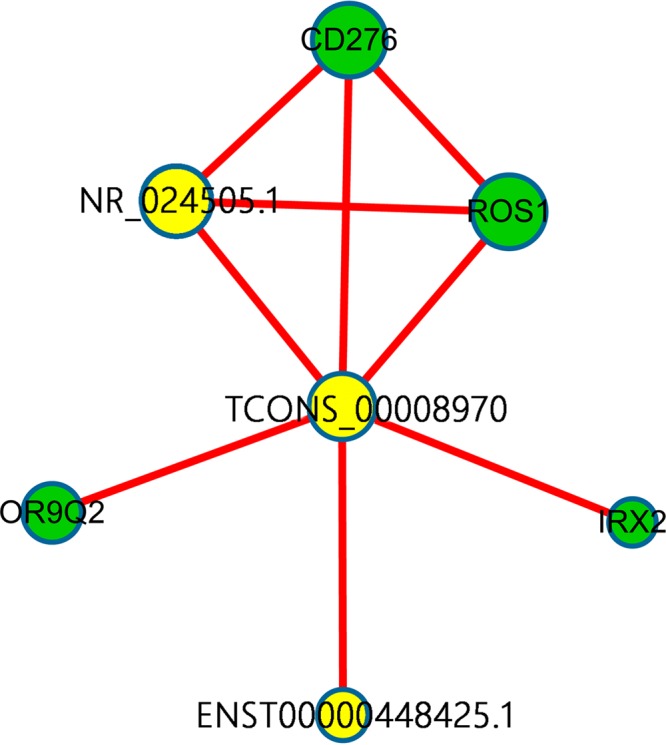
LncRNA–mRNA network was constructed to present the correlation analysis between the differentially expressed lncRNAs and mRNAs Yellow node represents the lncRNAs and green node represents the target mRNAs. The red outgoing link represents up-regulation, the size of each circle represents the significance of the correlation.

To determine which lncRNAs and mRNAs play critical roles in SAP progression, we constructed co-expression networks of the differentially expressed correlated lncRNAs and mRNAs. As shown in Figure 7, there were three source lncRNAs (NR_024505.1, ENST00000448425.1, and TCONS_00008970) indicated by yellow cycles) that were correlated with differential expression (down- or up-regulation) of corresponding genes by the criterion that coefficients obtained with no less than 0.99. The details of the source lncRNAs and their corresponding genes are described in Table 4. According to the correlated references and other studies, ROS1 [22] is a protein coding gene which related to transferase activity, transferring phosphorus-containing groups and protein tyrosine kinase activity, and is associated with ERK signalling and Akt signalling pathways. CD276 [23] is related to signalling receptor binding and NF-κB signalling pathway.

**Table 4:** LncRNA–mRNA network analysis

Source	Target	Correlation	*P*	Target. Gene symbol
NR_024505.1	A_23_P70278	0.993	8.252e-09	ROS1
	A_33_P3222917	0.995	3.9524e-09	CD276
ENST00000448425.1	p22630	0.997	5.101e-10	-
TCONS_00008970	A_33_P3222917	0.995	1.8592e-09	CD276
	A_33_P3297562	0.990	3.9582e-08	IRX2
	A_33_P3358779	0.990	3.9284e-08	OR9Q2
	A_23_P70278	0.992	2.1285e-08	ROS1
	p42653_v4	0.995	2.7186e-09	-

### qRT-PCR validation

**Figure 8:**
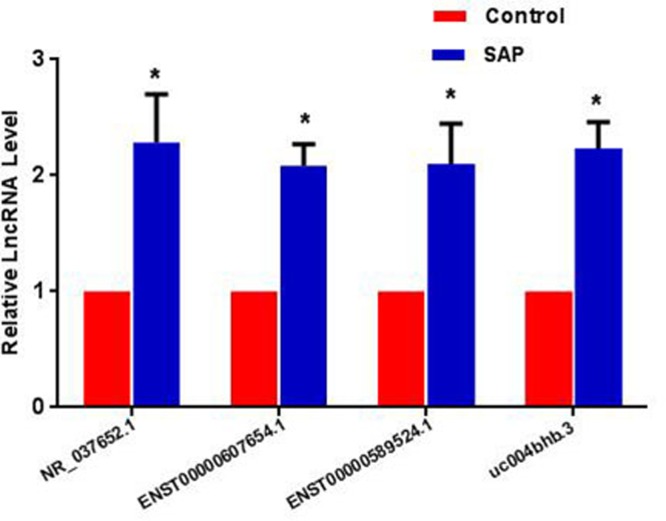
Validation data of the four strictly screened up-regulated lncRNAs by qRT-PCR in 50 SAP patients and 50 controls *P<0.05 compared with controls.

To validate the microarray data, four up-regulated lncRNAs (NR_037652.1, ENST00000607654.1, ENST00000589524.1 and uc004bhb.3) were selected based on the functional co-expression networks, the signal value and the validation of designed primers. A description of the four strictly screened lncRNAs is presented in Table 5. The four lncRNAs were all up-regulated between 50 Uyghur SAP patients and 50 matched controls, which were consistent with the microarray data (Figure 8). The characteristics of the 100 validated participants are shown in Table 6. For an in-depth understanding of the four lncRNAs, the target genes are mainly based on correlation of at least 0.8, not only the possible function (Supplementary Figure S1).

**Table 5:** The description of the four strictly screened lncRNAs

lncRNA ID	*P*	Fc	Regulation	Probe	Chrom	Start	End	Class
NR_037652.1	0.018	2.286	Up	p33678	15	80191181	80216096	Antisense
ENST00000607654.1	0.015	2.083	Up	p37346	2	220550102	220552067	Unkown
ENST00000589524.1	0.000	2.101	Up	p8706	19	36440385	36442421	Intergenic
uc004bhb.3	0.001	2.124	Up	p26507	9	116075501	116077893	Divergent

**Table 6:** Clinical characteristics of 50 Uyghur SAP patients and 50 Uyghur controls

Characteristics	Controls (*n*=50)	SAP patients (*n*=50)
Age, years	52.1 ± 6.14	51.6 ± 5.83
Gender (male/female)	38/12	38/12
BMI	24.7 ± 3.02	25.4 ± 3.26
Hypertension (Yes/No)	3/47*	10/40*
Diabetes (Yes/No)	7/43	11/39
Arrhythmia (Yes/No)	2/48	5/45
Medication (Yes/No)	2/48	8/42
Smoking (Yes/No)	8/42*	20/30*
Drinking (Yes/No)	3/47	6/44
SBP, mmHg	123.6 ± 14.31	116.9 ± 20.35
DBP, mmHg	83 ± 9.75	79.6 ± 16.19
Heart rate, beats per minute	71 ± 7.14	81 ± 9.51
Serum creatinine, μmol/l	80.4 ± 10.41	77.44 ± 17.05
FPG, mmol/l	4.94 ± 0.26	5.36 ± 1.17
TG, mmol/l	1.79 ± 0.5*	2.74 ± 2.59*
TC, mmol/l	4.86 ± 0.82	4.17 ± 0.69
LDL, mmol/l	3.49 ± 0.91	2.97 ± 0.58
Serum calcium, mmol/l	2.24 ± 0.07	1.94 ± 0.68

The data are presented as the mean ± SD. Abbreviations: BMI, body mass index; DBP, diastolic blood pressure; FPG, fasting plasma glucose; LDL, low-density lipoprotein; SBP, systolic blood pressure; TC, total cholesterol; TG, triglyceride. *P<0.05 compared with controls.

## Discussion

Cardiovascular disease (CVD) is the leading cause of death and disability worldwide, and SAP is the most common form of heart disease. Patients may have chest pain or breathlessness on exertion, but not at rest. It is important to reduce the occurrence of SAP, due to such reductions that have been shown to reduce heart attack and death rates [24]. Meanwhile, it is critical to detect SAP early and select appropriate treatments. Notably, recent genetic studies have demonstrated that the genetic component plays an important role in the development of CVD.

LncRNAs have been found to be an important genetic component of the genome regulatory network and to play important roles in disease processes [25]. Many efforts have been made to identify diagnostic and prognostic lncRNA biomarkers in various human cancers [26–28]. To date, there has been a lack of investigation into the utility of lncRNAs as biomarkers for the early diagnosis of SAP. Several profile-based studies have identified altered lncRNA expression during the initiation and progression of MI. For example, Zangrando et al. [29] used microarray analysis on MI mice to investigate the roles of differentially expressed lncRNAs in left ventricular remodelling, and Qu et al. [30] identified 545 deregulated lncRNAs involved in cardiac fibrogenesis induced by MI using microarray analysis. Furthermore, two other studies constructed dysregulated lncRNA–mRNA co-expression networks to investigate the functional roles of lncRNAs in MI and identified some key lncRNA candidates [31,32], emphasising the potential of lncRNAs to be used as biomarkers for the early diagnosis of MI.

Following the exploration in expression pattern of lncRNAs in MI [8], we investigated the genome-wide expression profile of lncRNAs in Uyghur ethnicity SAP in Xinjiang. A total of 1871 up- and 231 down-regulated lncRNAs were identified to be significantly and differentially expressed in SAP patients, accordingly in mRNAs level, 795 mRNAs were up-regulated and 554 mRNAs were down-regulated when compared with the healthy matched controls. To predict the potential functions of the lncRNAs, their co-expressed mRNAs were subjected to GO annotation and KEGG analysis. The most enriched GO annotation corresponding to the dysregulation of mRNAs related to SAP was the regulation of antioxidant activity. The significantly enriched disease terms included cholesterol (HDL), thrombophilia, pulse, calcium, coronary disease and metabolism. Among the top 20 aberrantly expressed lncRNAs, the annotation results of their co-expressed mRNAs showed that the most significantly related pathways were the NF-κB signalling pathway, apoptosis and the p53 signalling pathway. According to previous study, it has been proved that NF-κB signalling pathway [33,34] and p53 signalling pathway [34,35] serve not only an essential role in CHD, but also a significant effect on inflammatory response, oxidative stress and apoptosis. In order to reveal the co-expressions, we specifically picked out three lncRNAs (NR_024505.1, ENST00000448425.1 and TCONS_00008970), mainly due to the potential function of their co-expressed mRNAs (ROS1, CD276, OR9Q2 and IRX2). So far, several researches [36,37] have presented that ROS1 is correlated with CVDs, especially MI and sudden cardiac death. Anzalone et al. [38] has proved that CD276 is associated with heart failure, and IRX2 [39] undertakes an essential role in the development of heart. Importantly, previous studies [22,23] have revealed that the potential mechanisms of the mRNAs are related to NF-κB signalling pathway, ERK signalling pathway and Akt signalling pathways in cancers. These findings suggest that coordinated patterns of lncRNAs and their co-expressed mRNAs might be involved in the development of SAP. We speculated that these mRNAs may participate in the occurrence and development of SAP in the Uyghur population by regulating the three selected lncRNAs in the present study. However, this hypothesis needs to be further investigated.

Some lncRNAs have been reported to be biomarkers for the diagnosis of CVD; for example the lncRNA PCA3 has been reported to be a biomarker for severe left ventricular remodelling after MI [40], the circulating levels of lincRNA-P21 [41] are markedly increased in atherosclerosis and may be important in its pathogenesis, and the lncRNA OTTHUMT00000387022 [42] has been reported as a biomarker in coronary artery disease. However, these previous studies did not involve different ethnicities. Compared with these reports, our previous research [8] aimed to identify novel biological biomarkers which may have a potential in better management and stratification of Uyghur AMI patients. The result not only presented the expression profile of lncRNA using the same microarray, but also identified that three non-reported novel lncRNAs (ENST00000416860.2, ENST00000421157.1 and TCONS_00025701) were decreased in AMI patients, which had served as potential biomarkers. In order to illustrate whether lncRNAs can provide new biomarkers for Uyghur SAP patients, we have searched some lncRNAs as follows: firstly, we explored the primary data, and selected the functionally related mRNA through GO, KEGG and Disease enrichment (the main function of CVDs, the level of lipid and inflammation pathway). Secondly, we focussed on the co-expression network and chose the closely related lncRNAs. Finally, we selected according to the signal value and primers which must be designed and validated successfully. As a result, we singled out the four lncRNAs (NR_037652.1, ENST00000607654.1, ENST00000589524.1 and uc004bhb.3), and found that the levels of the four lncRNAs were elevated in Uyghur SAP patients. These results indicate that these differentially expressed lncRNAs may be potential biomarkers for the diagnosis of SAP in the Uyghur population. Our results provide a supplementary data in this field.

Some limitations of our study should be acknowledged. First, the present study used relatively few lncRNA probes compared with the number of known lncRNAs in some databases because the lncRNA expression profiles were obtained based on the Agilent Human lncRNA + mRNA Array V4.0. Second, the patients in the present study were from one hospital, not a multi-centre and larger scale study. Further verification from different areas and different races should be carried out to study the functional roles of these candidate lncRNA biomarkers in SAP. Finally, we forecast the function of lncRNA through high-throughput microarray and complex bioinformatics analysis. In the follow-up stage, we need to investigate the biological significances in model systems or cell lines which may contribute to understanding of the pathological mechanism of SAP.

In conclusion, our study examines the expression profile of lncRNA and mRNA in PBMCs from Uyghur SAP patients in comparison with matched controls using microarray. The results provide previously unreported bioinformation on Uyghur SAP patients, based on genomic-wide lncRNA expression and corresponding mRNA expression. These findings, on the one hand, provide useful bioinformation which may have a potential role in the development of SAP; on the other hand, emphasised the potential of lncRNAs to be used as biomarkers for the early diagnosis of SAP.

## Supporting information

**Supplementary Figure S1:** **(A)** LncRNA-mRNA network was constructed between the NR_037652.1 and mRNAs. **(B)** LncRNA-mRNA network was constructed between the ENST00000607654.1 and mRNAs. **(C)** LncRNA-mRNA network was constructed between the ENST00000589524.1 and mRNAs. **(D)** LncRNA-mRNA network was constructed between the uc004bhb.3 and mRNAs.

**Supplementary Material:** 

## Abbreviations

AMI: acute myocardial infarction
CHD: coronary heart disease
CVD: cardiovascular disease
Fc: fold-change
GO: Gene Ontology
HDL: high density lipoprotein
KEGG: Kyoto Encyclopedia of Genes and Genomes
LDL: low density lipoprotein
lncRNA: long non-coding RNA
PBMC: peripheral blood mononuclear cell
qRT-PCR: quantitative real-time polymerase chain reaction
SAP: stable angina pectoris
SSC: saline sodium citrate
