# Supplementary material for: Expression profiles and potential functions of long non-coding RNA in stable angina pectoris patients from Uyghur population of China
Source: Biosci Rep. 2019 Sep 3;39(9):BSR20190364. doi: 10.1042/BSR20190364 (PMC6722491; doi:10.1042/BSR20190364)
Supplement: Supplementary file 1 [file bsr20190364_Supp1.pdf]

Supplementary Figure 1. (A) LncRNA-mRNA network was constructed between the NR_037652.1 and mRNAs. **(B)** LncRNA-mRNA network was constructed between the ENST00000607654.1 and mRNAs. **(C)** LncRNA-mRNA network was constructed between the ENST00000589524.1 and mRNAs. **(D)** LncRNA-mRNA network was constructed between the uc004bhb.3 and mRNAs.

(A)

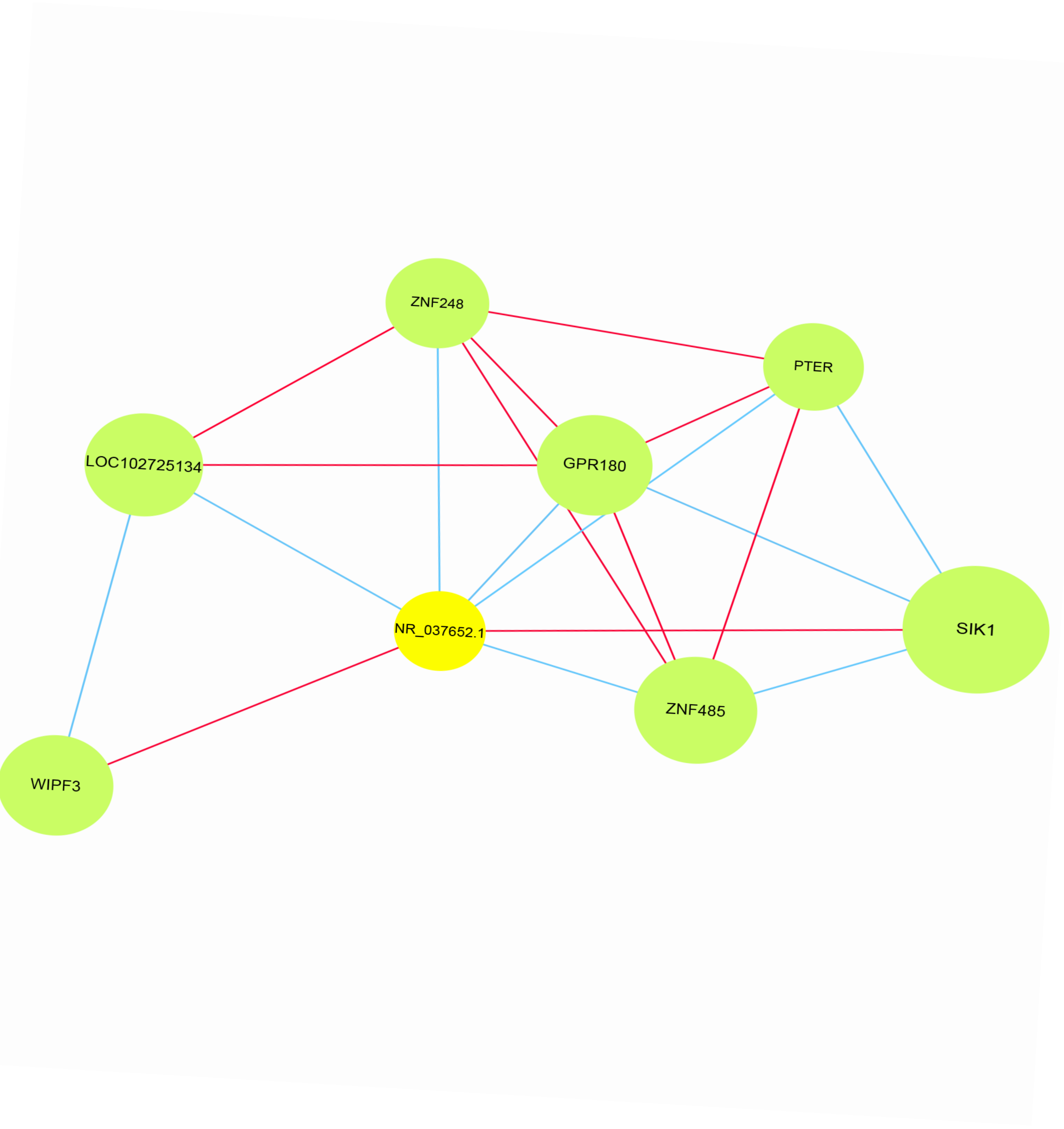
ZNF248
PTER
LOC102725134
GPR180
NR_037652.1
SIK1
ZNF485
WIPF3

(B)

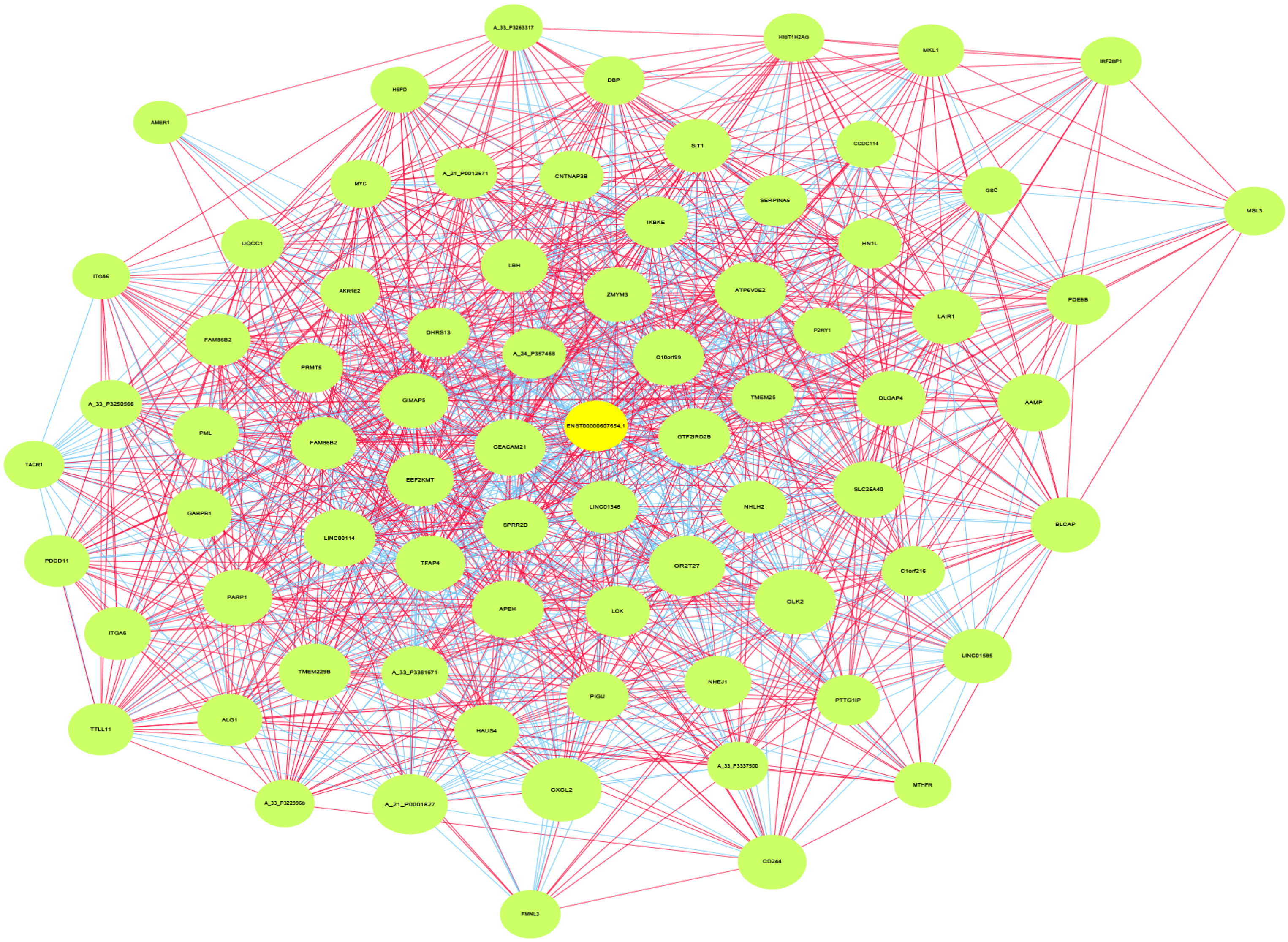

A_33_P3263317
HIST1H2AG
MKL1
IRF2BP1
DBP
H6PD
AMER1
SIT1
CCDC114
A_21_P0012571
MYC
CNTNAP3B
SERPINA5
GSC
MSL3
IKBKE
UQCC1
HN1L
ITGA6
LBH
AKR1E2
ZMYM3
ATP6V0E2
PDE6B
LAIR1
P2RY1
FAM86B2
DHRS13
A_24_P357468
C10orf99
PRMT5
TMEM25
DLGAP4
AAMP
A_33_P3250566
GIMAP5
ENST00000607654.1
PML
FAM86B2
GTF2IRD2B
CEACAM21
TACR1
EEF2KMT
SLC25A40
LINC01346
NHLH2
BLCAP
GABPB1
SPRR2D
LINC00114
PDCD11
TFAP4
OR2T27
C1orf216
CLK2
PARP1
APEH
LCK
ITGA6
LINC01585
TMEM229B
A_33_P3381671
NHEJ1
PIGU
PTTG1IP
TTLL11
ALG1
HAUS4
A_33_P3337500
MTHFR
CXCL2
A_33_P3229968
A_21_P0001827
CD244
FMNL3

(C)

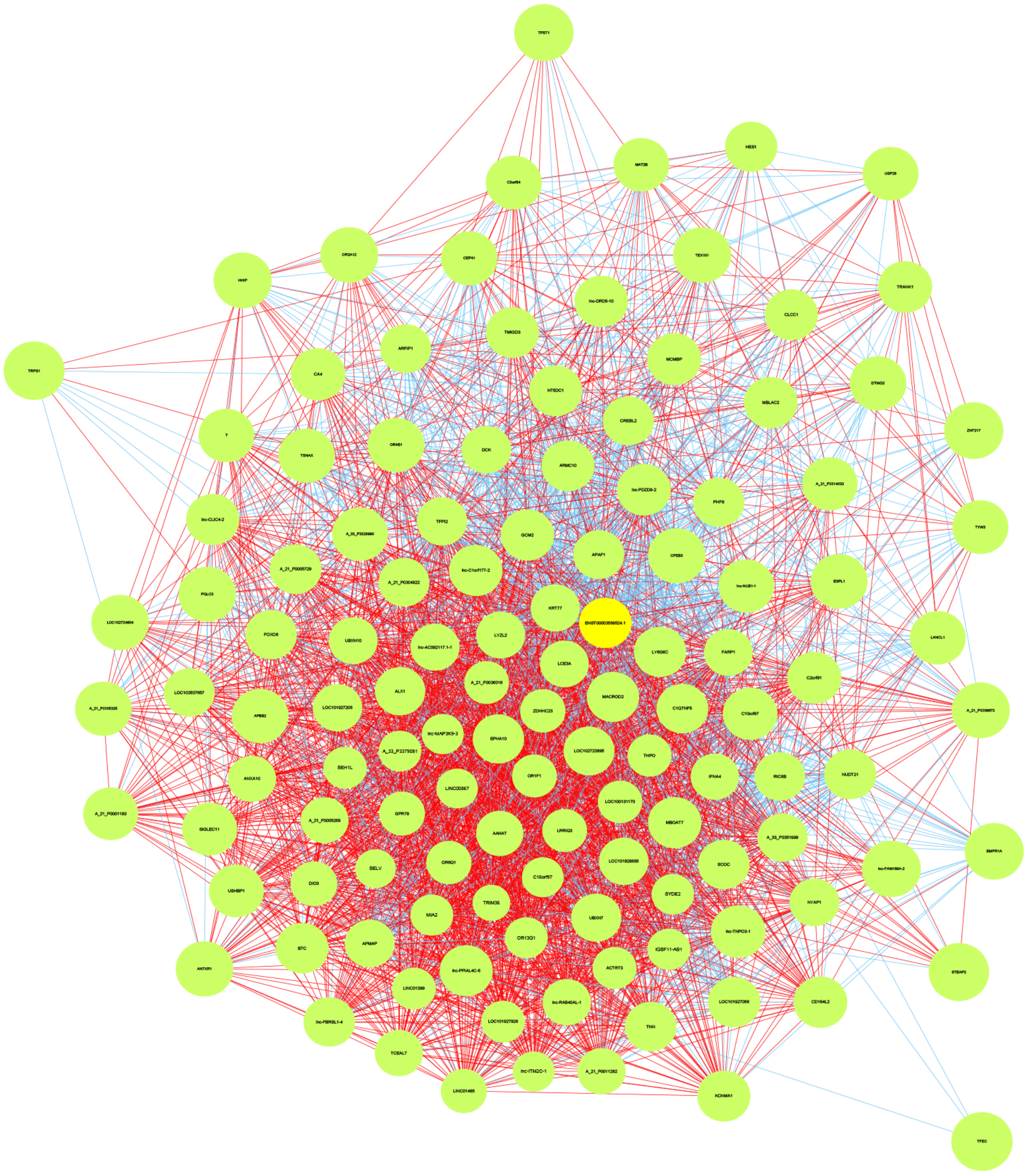

(D)

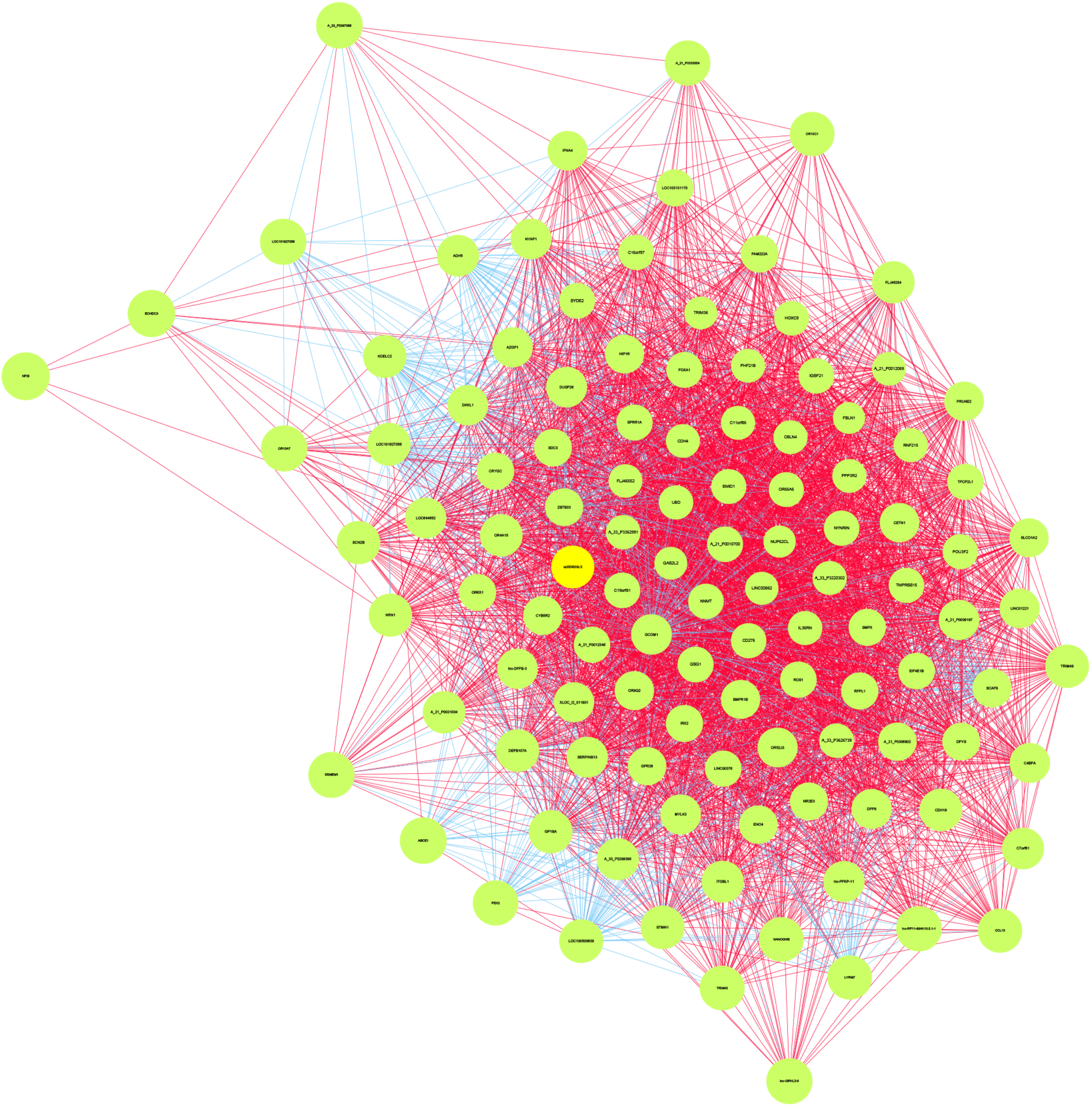

Supplementary Table S1. PCR primers of four selected lncRNAs.

Gene Name	Forward Primer	Reverse Primer	Hybridization Temperature (℃)
NR_037652.1	GAGGAGGCGGTTCATCTGTG	GAAGAGCCGGAGGAAAGAGG	60
ENST00000607654.1	AGGCACCTCTTATGCTTGCT	TAATGGGTAGCCAGGGGTCA	60
ENST00000589524.1	CATGTGTCTTGGGGTGGCTA	TGCAAAGAGCTGCAAAAGCG	60
uc004bhb.3	CAGGGACAGTCGTAGCATGT	TACTTTCCTTCTCCTGGCAGC	60
